# Diagnostic and prognostic potential of exosome non-coding RNAs in bladder cancer: a systematic review and meta-analysis

**DOI:** 10.3389/fonc.2024.1336375

**Published:** 2024-03-04

**Authors:** Yani Chen, Kesong Shi, Xinyao Fu, Hua Guo, Tian Gao, Haiquan Yu

**Affiliations:** State Key Laboratory of Reproductive Regulation and Breeding of Grassland Livestock, School of Life Sciences, Inner Mongolia University, Hohhot, Inner Mongolia, China

**Keywords:** bladder cancer, non-coding RNAs, prognosis, diagnosis, meta-analysis

## Abstract

**Background:**

Bladder cancer stands as the predominant malignant tumor in the urological system, presenting a significant challenge to public health and garnering extensive attention. Recently, with the deepening research into tumor molecular mechanisms, non-coding RNAs (ncRNAs) have emerged as potential biomarkers offering guidance for the diagnosis and prognosis of bladder cancer. However, the definitive role of ncRNAs in bladder cancer remains unclear. Hence, this study aims to elucidate the relevance and significance of ncRNAs through a Meta-analysis.

**Methods:**

A systematic meta-analysis was executed, including studies evaluating the diagnostic performance of ncRNAs and their associations with overall survival (OS) and disease-free survival (DFS). Key metrics such as hazard ratios, sensitivity, specificity, and diagnostic odds ratios were extracted and pooled from these studies. Potential publication bias was assessed using Deeks’ funnel plot, and the robustness of the results was ascertained through a sensitivity analysis.

**Results:**

Elevated ncRNA expression showed a positive correlation with improved OS, evidenced by a hazard ratio (HR) of 0.82 (95% CI: 0.66-0.96, P<0.001). Similarly, a significant association was observed between heightened ncRNA expression and DFS, with an HR of 0.86 (95% CI: 0.73-0.99, P<0.001). Diagnostic performance analysis across 17 articles yielded a pooled sensitivity of 0.76 and a specificity of 0.83. The diagnostic odds ratio was recorded at 2.71, with the area under the ROC curve (AUC) standing at 0.85.

**Conclusion:**

Exosome ncRNAs appear to possess potential significance in the diagnostic and prognostic discussions of bladder cancer. Their relationship with survival outcomes and diagnostic measures suggests a possible clinical utility. Comprehensive investigations are needed to fully determine their role in the ever-evolving landscape of bladder cancer management, especially within the framework of personalized medicine.

## 1 Introduction

Bladder cancer (BCa), a leading urological malignancy, is characterized by its escalating global incidence and mortality (1–3). Despite advancements in diagnostic and therapeutic interventions, the 5-year overall survival rate for BCa remains suboptimal, primarily due to its predilection for swift recurrence and aggressive metastasis (4, 5). In this evolving landscape, the emergence of molecular biomarkers, especially exosome non-coding RNAs (ncRNAs), offers a promising frontier. Such markers are poised to revolutionize patient management by facilitating treatments tailored to individual molecular signatures, thereby enhancing therapeutic efficacy and survival outcomes (6–8). As we delve deeper into the molecular intricacies of BCa, the imperative to harness these biomarkers becomes paramount, heralding an era dominated by personalized medicine with promising prognostic implications.

Recent molecular biology breakthroughs have underscored the crucial involvement of ncRNAs in diverse cellular pathways, accentuating their role in tumorigenesis and the intricate cascade of cancer progression (9, 10). The vast realm of ncRNAs, which includes microRNAs (miRNAs), long non-coding RNAs (Lnc RNAs), and circular RNAs (circ RNAs), has become a nexus of intense research scrutiny (11–13). However, it’s their association with exosomes, nanoscale extracellular vesicles vital for intercellular communication, that is at the forefront of ongoing research (8, 14, 15). Unraveling the complexities of ncRNAs encapsulated in exosomes can potentially provide unprecedented insights into cancer biology, heralding novel therapeutic and diagnostic paradigms.

A growing corpus of literature has emphatically underscored ncRNAs as paramount molecular sentinels in the BCa diagnostic and prognostic arena (6, 16, 17). he differential expression patterns of ncRNAs in oncogenic pathways earmark them as potent biomarkers (18). Yet, there’s a palpable discord concerning the conclusive prognostic efficacy of exosome ncRNAs (6–8, 14–17, 19–21). Such variances stem from disparate study designs, diverse methodologies, sample sizes, and demographic intricacies, thereby muddying the interpretative waters.

This study aims to systematically investigate the potential prognostic value of exosome ncRNA in bladder cancer patients by conducting a comprehensive meta-analysis that integrates the results of multiple studies. Meta-analysis, through the aggregation of a substantial volume of data, serves to identify and strengthen the consistent associations between exosome ncRNA and bladder cancer prognosis, which may not have been fully revealed by individual studies. Furthermore, by addressing the heterogeneity and methodological differences present in existing research, this study seeks to provide more comprehensive and reliable evidence to support the potential application of exosome ncRNA in the assessment of bladder cancer prognosis.

## 2 Materials and methods

### 2.1 Search strategies

A computerized search was conducted in the databases of PubMed, Web of Science, EMBASE, and Cochrane Library for literature related to ncRNA and bladder cancer. The search strategy combined both Mesh terms and free-text words. The principal keywords employed were “Bladder Cancer”, “Non-coding RNA”, “Prognosis”, and “Survival Rate”. To refine the search results, only English language articles were considered, and the search date was updated to September 1, 2023. This search strategy has been registered with the PROSPERO (https://www.crd.york.ac.uk/prospero/) under the registration number CRD42023454417.

### 2.2 Inclusion and exclusion criteria

Inclusion criteria consisted of: (1) Studies focusing on populations diagnosed with bladder cancer; (2) Research encompassing exosome non-coding RNA; (3) Studies presenting outcomes on diagnostic accuracy and prognostic indices, such as Overall Survival (OS) or Disease-Free Survival (DFS).

Exclusion criteria were: (1) Commentaries, symposium abstracts, case narrations, and expert reviews; (2) Duplicate studies; (3) Studies missing a control group (non-BCa); (4) Studies with significant data absence or unextractable data.

### 2.3 Literature screening and data extraction

Upon completing the literature search, two researchers carried out systematic literature reviews and data collation to ensure accuracy and consistency. An initial screening was conducted based on article titles and abstracts to exclude manuscripts that were clearly inconsistent with the inclusion criteria or met the exclusion criteria. Subsequently, a thorough reading and assessment of the preliminarily selected literature were performed. Key data were then extracted from the finalized studies, which included research type, sample size, diagnostic precision, and specificity of ncRNAs, as well as prognostic indicators such as OS and DFS Hazard Ratios (HR) along with their respective 95% confidence intervals (CI). In cases of discrepancies or disputes during the literature screening and data extraction processes, resolutions were sought through consultations with a third author.

### 2.4 Quality assessment

In an evaluation of the incorporated literature, we leveraged the Newcastle-Ottawa Scale (NOS) as our metric of discernment (22). The NOS systematically scrutinizes studies across three pivotal axes: selection, comparability and outcomes. Pp

Studies can accrue a zenithal score of 9 points, with elevated scores emblematic of paramount research caliber. To obviate subjective predilections and bolster the assessment’s rigor, this critical examination was helmed by two discrete authors.

### 2.5 Statistical analysis

Statistical analyses within this investigation were executed using the Stata 13 software. Initially, pertinent metrics, including sample sizes, counts of negatives, and positives, were extracted from each encompassed study, facilitating the computation of true positives (TP), false positives (FP), true negatives (TN) and false negatives (FP). Armed with this data, we deduced the sensitivity, specificity, positive predictive value, negative predictive value, and the diagnostic odds ratio (DOR). To gauge the cumulative effect magnitude of extracellular non-coding RNAs on bladder cancer prognosis, we amalgamated the HRs and their 95% CIs for OS and DFS from the assimilated studies. The *I²* statistic was harnessed to probe heterogeneity across the studies, with an *I²* exceeding 50% signaling pronounced heterogeneity. In the presence of marked heterogeneity, a random-effects model was adopted for meta-analysis; contrarily, a fixed-effects model was favored. Potential publication bias was discerned via funnel plot inspection. All inferential tests were bifurcated, with *P<0.05* demarcating statistical significance.

## 3 Results

### 3.1 Literature search and characteristics

**Figure 1:**
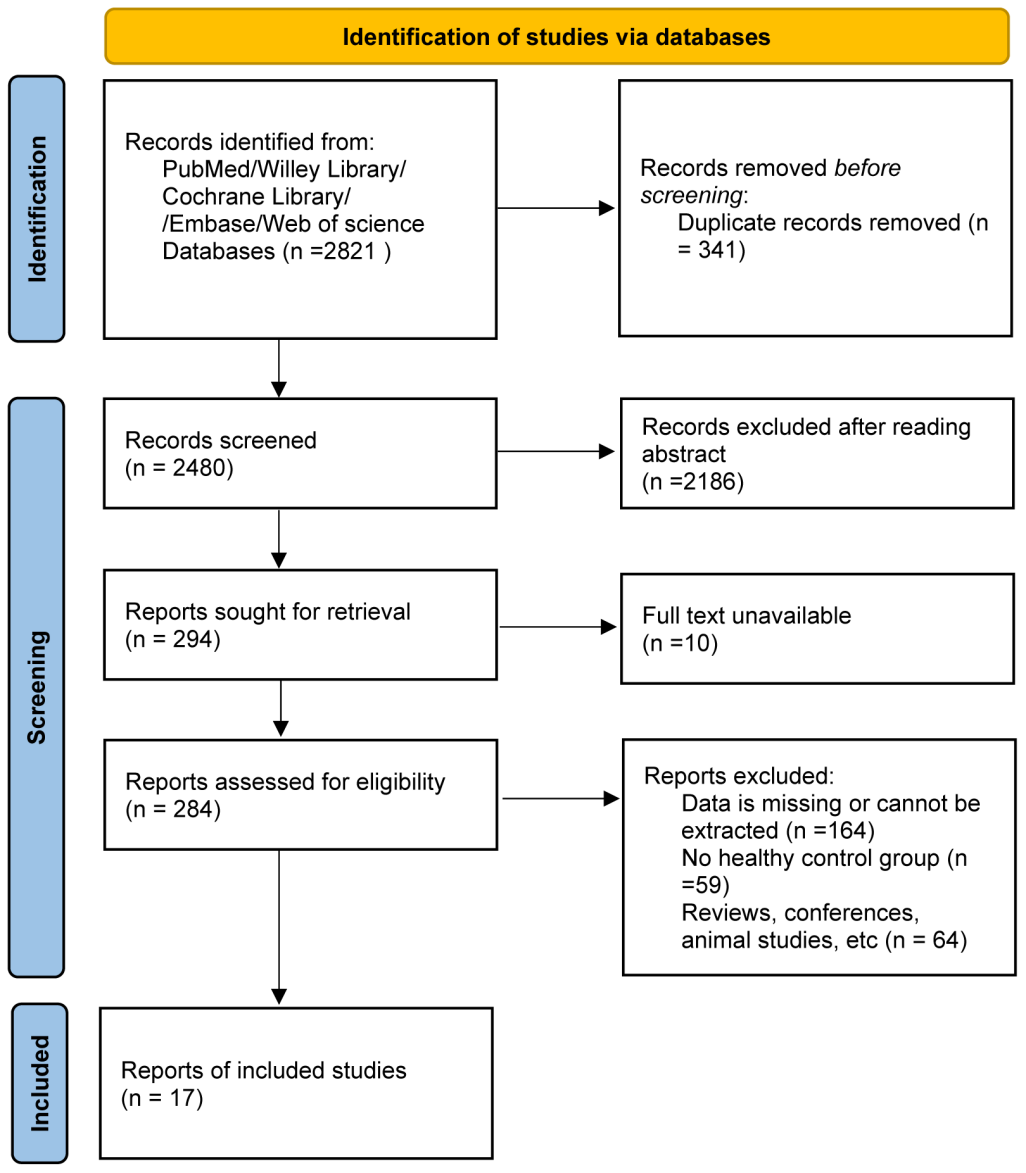
The flow diagram of the meta-analysis.

In our search process, we identified 2,821 pertinent publications. **Figure 1** provides a visual representation detailing the selection procedure and the rationale for exclusions. Of these, 284 studies were deemed suitable for an in-depth full-text review. Among them, 17 studies rigorously adhered to all inclusion criteria and were consequently selected for further analysis. The characteristics and specific attributes of these incorporated articles are comprehensively delineated in **Table 1** and further expounded upon in **Supplementary Tables S1, S2**. The quality assessment of all the included literatures met the standards set for this research, as demonstrated in **Supplementary Table S3**.

**Table 1:** Characteristics of included literatures in this meta-analysis.

First Author	Year	Country	Sample		Age (Year)		ncRNA type	Detection method	Classification of bladder cancer(Low/High)	Sample Type
			Control	BCa	Control	BCa				
Abbastabar (10)	2020	Iran	10	30	55.84 ± 11.96	57.4 ± 5.7	lncRNA	qRT-PCR	13/17	Urine
							lncRNA	qRT-PCR	13/17	Urine
Bian (9)	2022	China	43	43	70.05 ± 12.85	71.98 ± 9.88	lncRNA	qRT-PCR	9/34	Urine
Chen (23)	2020	China	120	206	/	/	lncRNA	qRT-PCR	/	Serum
Chen (19)	2022	China	94	128	/	/	lncRNA	qRT-PCR	20/108	Urine
Chen (24)	2021	China	242	242	/	/	lncRNA	qRT-PCR	/	Serum
Chen (25)	2018	China	31	70	/	/	miRNA	qRT-PCR	/	Serum
El-Shal (17)	2021	Egypt	28	51	58.4 ± 3.6	59.5 ± 3.2	miRNA	qRT-PCR	9/42	Urine
							miRNA	qRT-PCR	9/42	Urine
Güllü Amuran (16)	2020	Turkey	34	105	25~87	37~87	miRNA	qRT-PCR	16/89	Urine
Huang (6)	2021	China	80	80	47.4 ± 11.3	64.8 ± 12.5	lncRNA	qRT-PCR	35/45	Urine
							lncRNA	qRT-PCR	35/45	Urine
							lncRNA	qRT-PCR	35/45	Urine
Lin (26)	2021	China	51	53	52–69	54–68	miRNA	qRT-PCR	22/31	Urine
Wang (7)	2018	Iran	50	50	/	/	lncRNA	qRT-PCR	31/19	Serum
Xue (8)	2017		30	30	/	/	lncRNA	qRT-PCR	/	Serum
Yazarlou (14)	2018	Iran	49	59	61.28 ± 13.01	64.42 ± 15.53	lncRNA	qRT-PCR	20/38	Urine
							lncRNA	qRT-PCR	20/38	Urine
							lncRNA	qRT-PCR	20/38	Urine
							lncRNA	qRT-PCR	20/38	Urine
Zhan (15)	2018	China	80	80	/	/	lncRNA	qRT-PCR	50/30	Urine
							lncRNA	qRT-PCR	50/30	Urine
							lncRNA	qRT-PCR	50/30	Urine
Zhang (20)	2019	China	160	160	/	/	lncRNA	qRT-PCR	84/76	Serum
Zheng (27)	2021	China	112	210	/	/	lncRNA	qRT-PCR	/	Tissue
Zheng (21)	2018	China	50	60	67.0 ± 9.8	66.2 ± 10.7	lncRNA	qRT-PCR	35/15	Plasma

### 3.2 Association between ncRNAs expression and prognosis

#### 3.2.1 ncRNAs expression and OS

**Figure 2:**
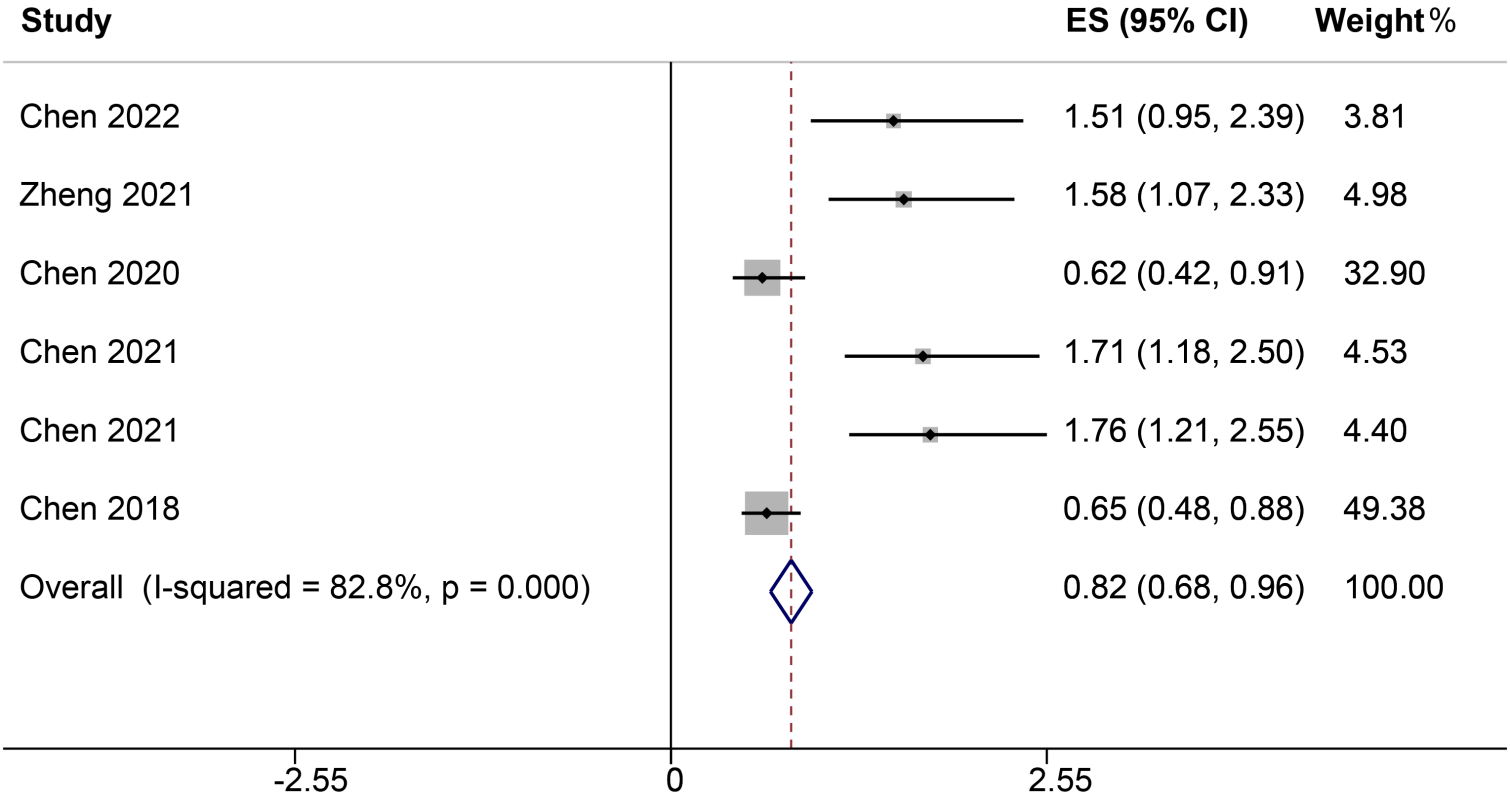
Forest plots of the relationship between ncRNA expression and OS.

Four studies, encompassing six different ncRNAs, reported on the relationship between low and high ncRNA expression levels and OS. Heterogeneity analysis revealed significant variability among the included publications, with an *I²* value of 82.8%. Due to this pronounced heterogeneity, a random-effects model was utilized for the analysis. The results indicated a significant correlation between elevated ncRNA expression and OS, with a HR of 0.82 (95% CI: 0.66-0.96, *P<0.001*, **Figure 2**).

#### 3.3.2 ncRNAs expression and RFS

**Figure 3:**
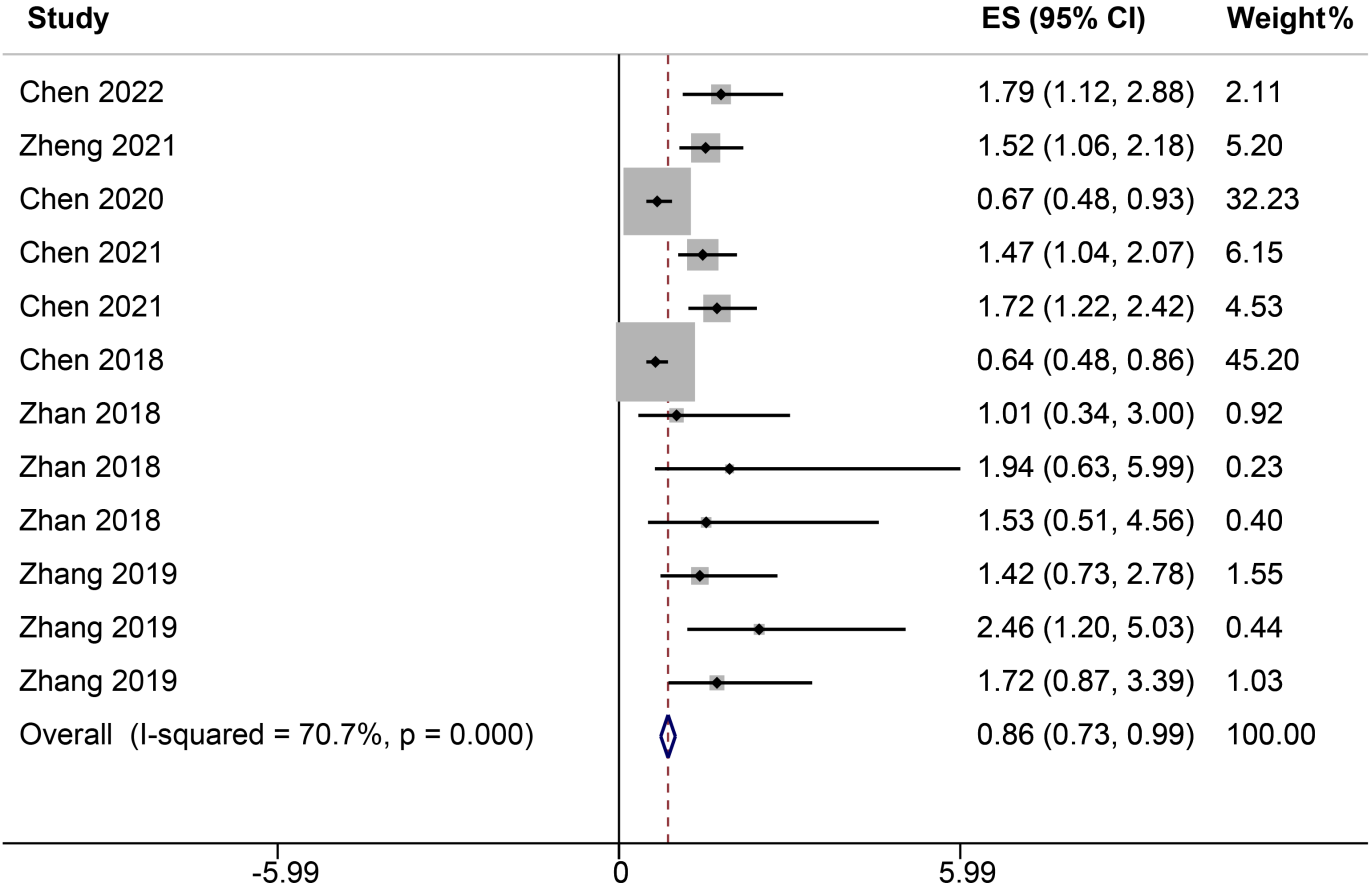
Forest plots of the relationship between ncRNA expression and DFS.

Seven studies, encompassing twelve distinct ncRNAs, delineated the association between varying ncRNA expression levels (low versus high expression) and DFS. A heterogeneity analysis highlighted substantial variability among the selected studies, evidenced by an *I²* value of 70.7%. Given this pronounced heterogeneity, the analysis was conducted using a random-effects model. The results reveal a significant correlation between elevated ncRNA expression and DFS, with a HR of 0.86 (95% CI: 0.73-0.99, *P<0.001*, **Figure 3**).

### 3.4 Meta-analysis of diagnostic ncRNA

#### 3.4.1 Diagnostic performance analysis of ncRNA

**Figure 4:**
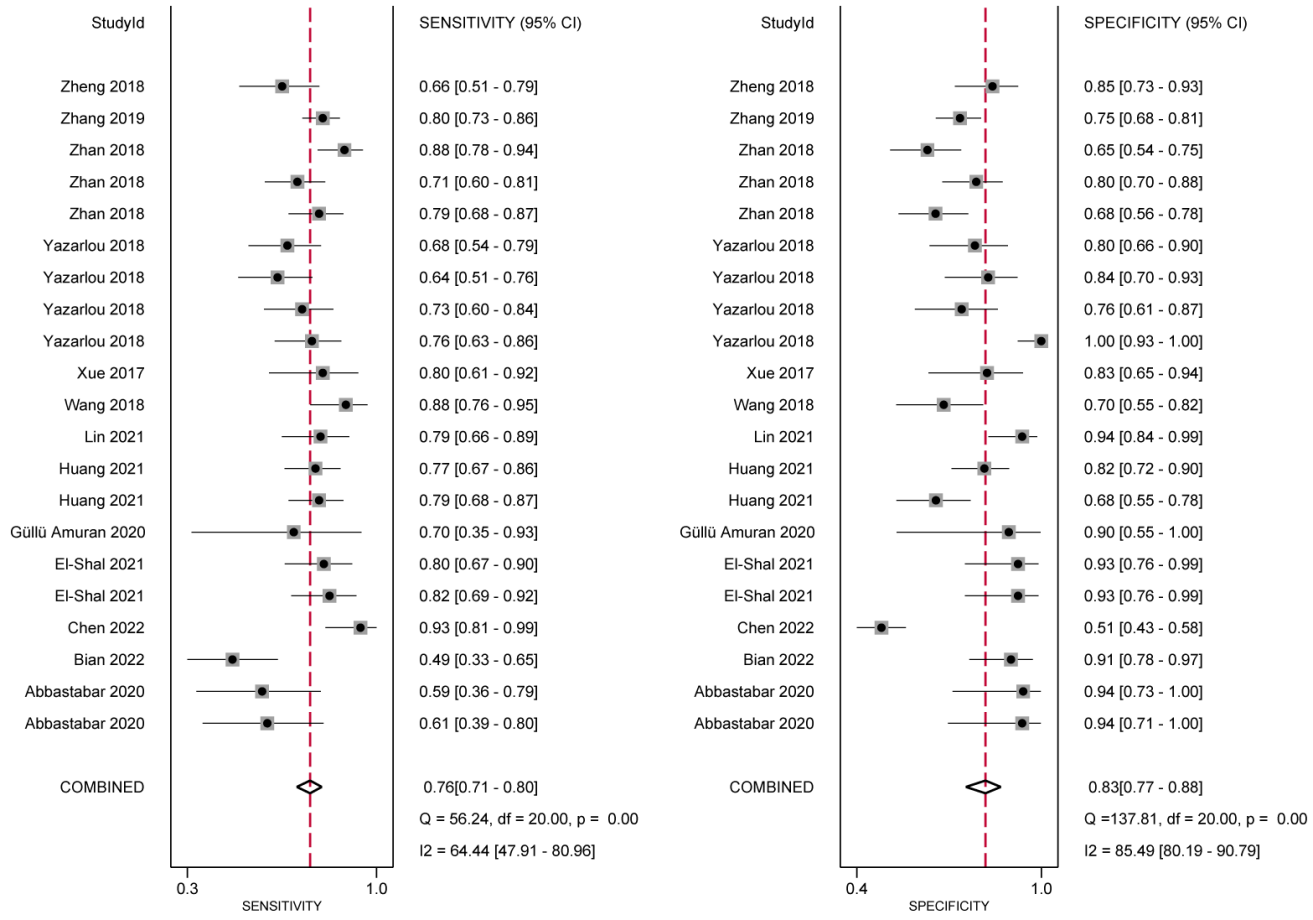
Forest plot of sensitivity and specificity of ncRNA expression for the diagnosis.

**Figure 5:**
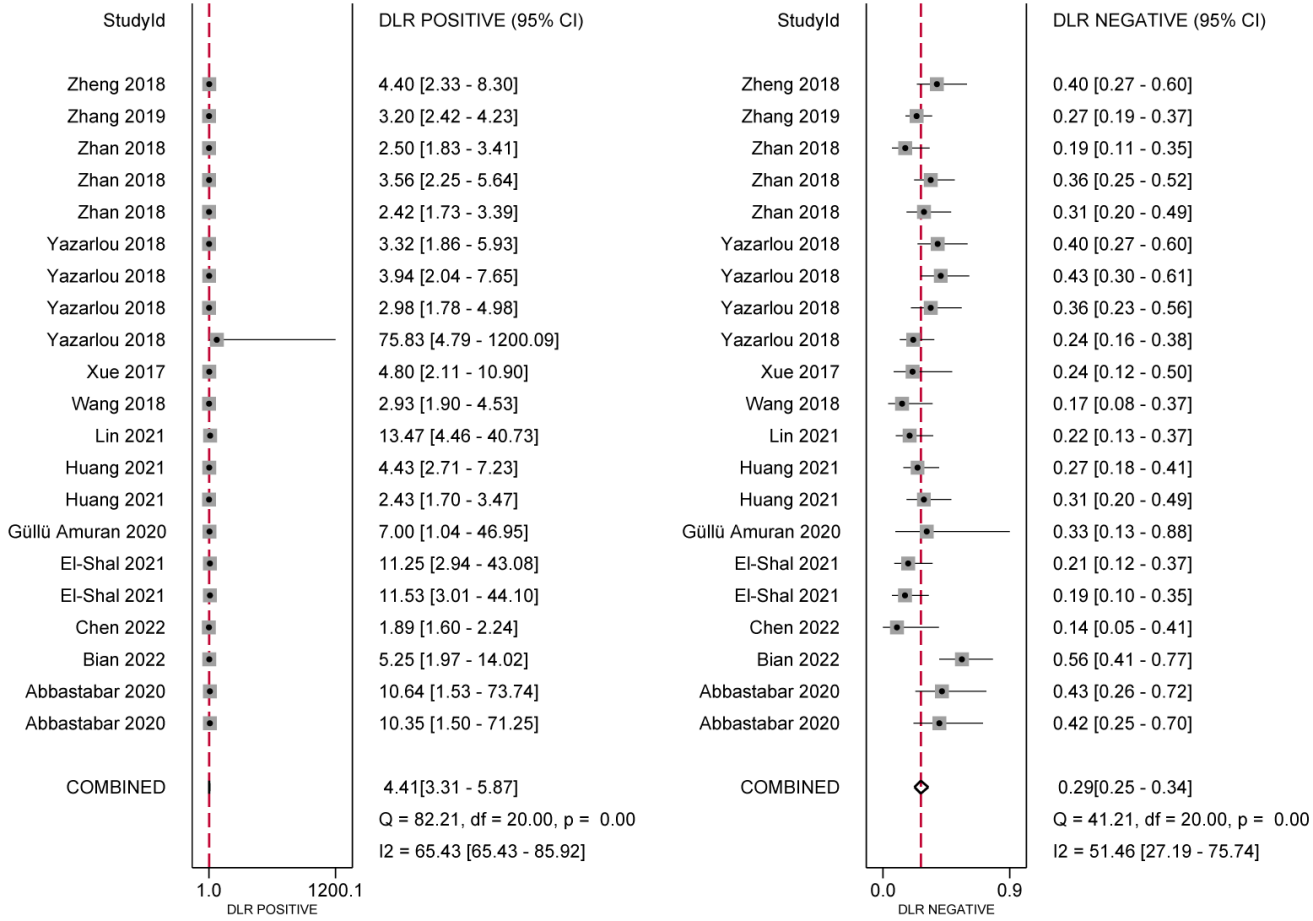
Positive Likelihood Ratio (PLR) and Negative Likelihood Ratio (NLR).

**Figure 6:**
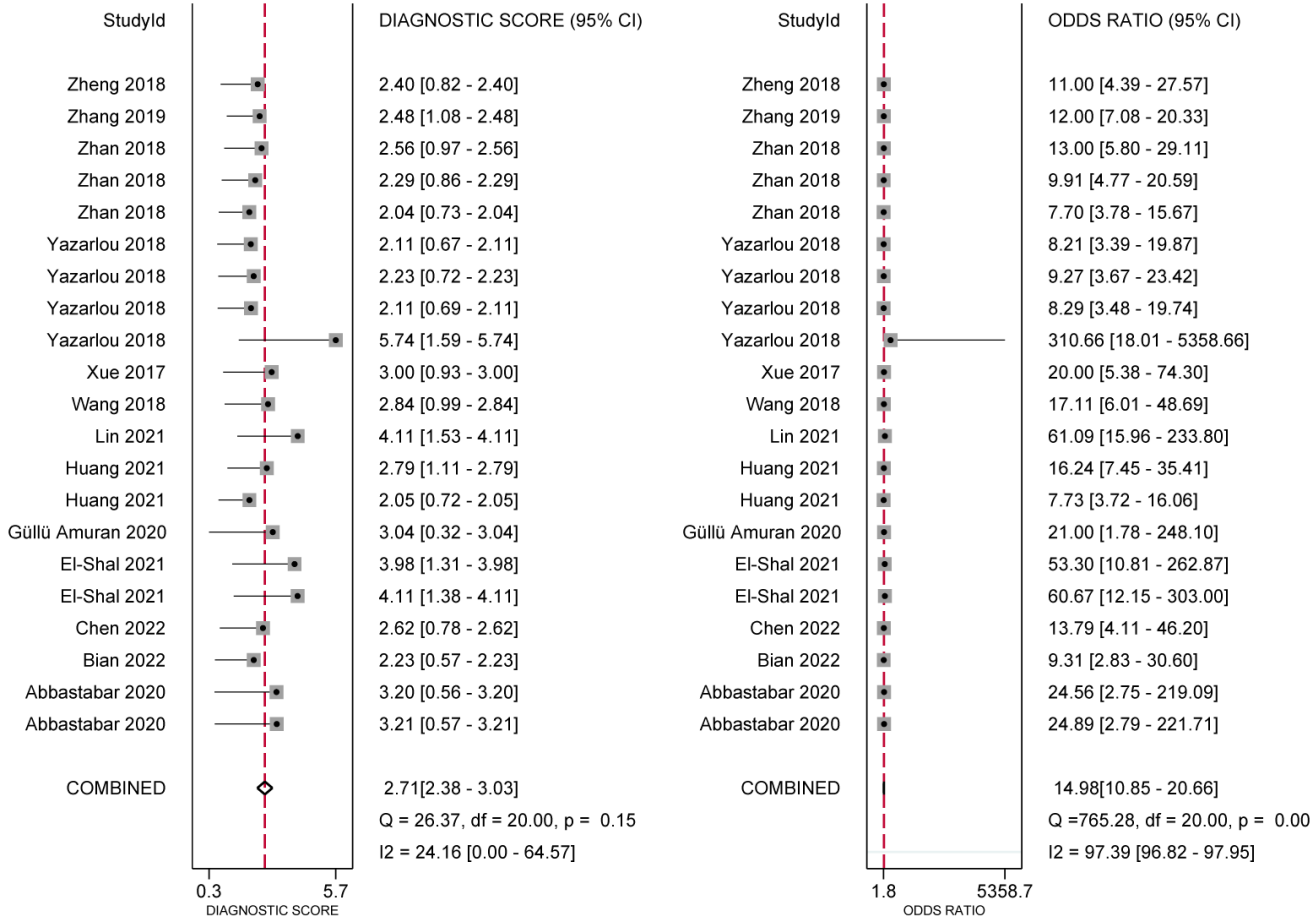
Diagnostic Odds Ratio and Diagnostic Score.

**Figure 7:**
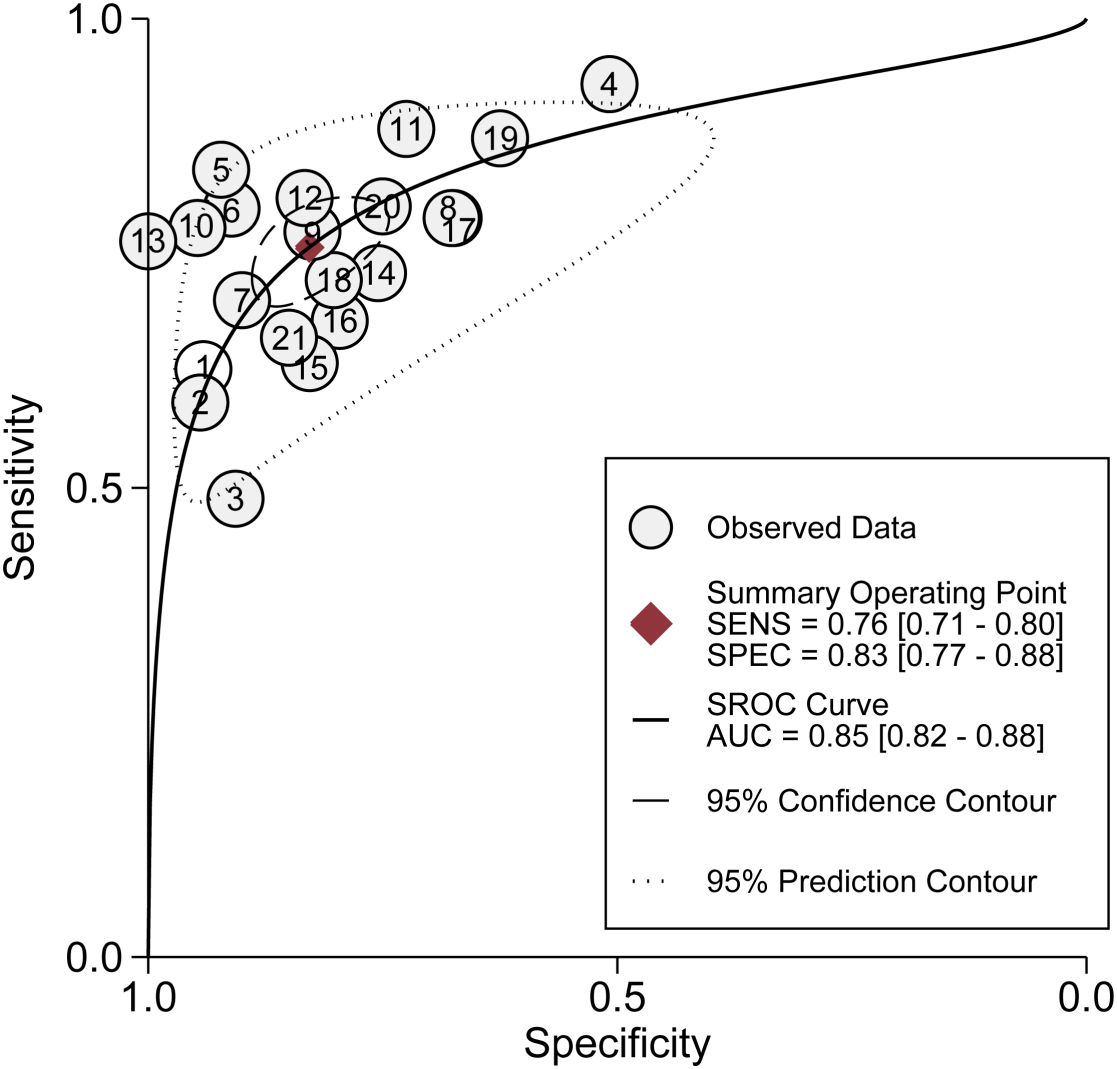
Summary receiving operating characteristic curves.

In the analysis of ncRNA diagnostic performance, a total of 17 articles involving 22 different ncRNAs were included. The meta-analysis yielded a pooled sensitivity of 0.76 (95% CI: 0.71–0.80; *I²=64.4%*) and specificity of 0.83 (95% CI: 0.77–0.88; *I² =85.49%*). The positive likelihood ratio (PLR) was 4.41 (95% CI: 3.31–5.87; *I² =65.43%*), while the negative likelihood ratio (NLR) was 0.29 (95% CI: 0.25–0.34; *I²=54.46%*). The diagnostic odds ratio was 2.71 (95% CI: 2.38–3.03; *I²=24.16%*), and the diagnostic score was 14.89 (95% CI: 10.85–20.66; *I²=97.39%*). Further confirmation of the high accuracy of ncRNA in bladder cancer diagnosis was obtained by drawing receiver operator characteristic (ROC) curves and calculating the area under the curve (AUC). The AUC was 0.85 (95% CI: 0.82–0.88), as shown in **Figures 4**–**7**.

#### 3.4.2 Prior probability and posterior probability

Using Fagan’s nomogram, we evaluated the influence of test outcomes on the posttest probability of diagnosis, based on a predefined pretest probability. The data underscores that ncRNA detection holds considerable diagnostic significance, substantially elevating the posttest probability for bladder cancer recognition, even starting from a relatively low pretest probability (**Supplementary Figure S1**). The scatter plot delineates a positive correlation between the primary variables (**Supplementary Figure S2**).

### 3.5 Publication bias

**Figure 8:**
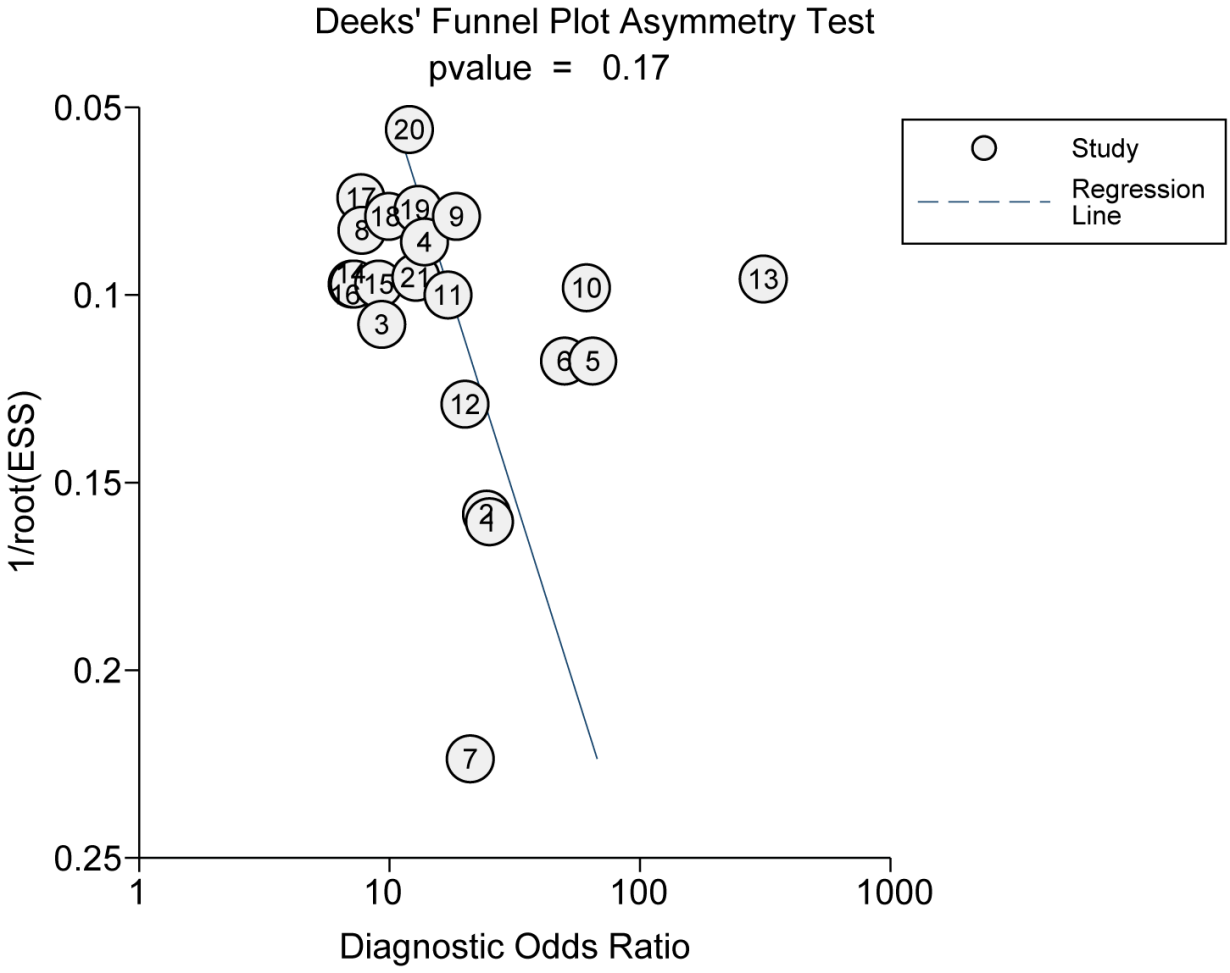
Deek’s Funnel Plot asymmetry test for assessing publication bias in the included studies.

Employing Deeks’ funnel plot, we undertook an assessment of potential publication bias among the studies incorporated. The nearly horizontal slope of the funnel plot, coupled with an associated p-value of 0.33, robustly suggests an absence of notable publication bias (**Figure 8**).

### 3.6 Sensitivity analysis

To ensure the robustness of our meta-analysis results, we employed a sensitivity analysis using the one-by-one omission method. The results demonstrated good stability after sequential removal (**Supplementary Tables S2, S3**).

## 4 Discussion

The molecular intricacies inherent to oncogenesis consistently highlight the profound influence of non-coding elements in cellular signaling and function (28, 29). Among these, exosome ncRNAs have surfaced as central orchestrators, coordinating myriad processes linked with tumor initiation, progression, and metastasis (24, 30). Among these, exosome ncRNAs have surfaced as central orchestrators, coordinating myriad processes linked with tumor initiation, progression, and metastasis (31, 32). Against this intricate backdrop, the present meta-analysis seeks to provide an exhaustive evaluation of the prognostic significance of exosome ncRNAs within bladder oncology. With meticulous amalgamation and interpretation, we delineate potential clinical trajectories these molecular agents might trace, suggesting a paradigm shift in diagnostic accuracy and therapeutic interventions for bladder cancer care.

### 4.1 Decoding the prognostic implications of ncRNA expression

The synthesis of various studies accentuates a pronounced correlation between ncRNA expression and survival rates in bladder cancer. This association, mirrored by consistency in DFS outcomes, underscores the cardinal role ncRNAs can play in the clinical evolution of bladder cancer (19, 23–25). This association, reiterated by the congruence in DFS outcomes, emphasizes the pivotal role ncRNAs might play in bladder cancer’s clinical course (15, 20, 25). However, the manifest heterogeneity across the included studies mirrors the multifaceted nature of bladder malignancies. Such heterogeneity may be attributed to a range of factors, including the genetic makeup of patient cohorts, tumor attributes, and methodological differences in ncRNA quantification. Addressing these disparities in subsequent research is paramount for a more integrated comprehension.

Recent years, exosomes have emerged as a non-invasive biomarker with immense potential in the early diagnosis and monitoring of bladder cancer. Exosomes are small vesicles released by cells into bodily fluids, capable of carrying a variety of molecules including ncRNAs, thus reflecting the biological state of their source cells (7, 33, 34). The use of exosomes for diagnosing bladder cancer presents distinct advantages, including their presence in various bodily fluids which facilitates easy and non-invasive sample collection, and the high stability of molecules within exosomes, contributing to the accuracy and reliability of detection (15, 20). However, this method also faces challenges, including the need for further optimization of exosome isolation and purification techniques to enhance efficiency and purity. Additionally, the current understanding of the relationship between specific ncRNAs within exosomes and the development of bladder cancer remains limited, necessitating more foundational and clinical research to deepen our knowledge.

### 4.2 Diagnostic potential: ncRNA’s forefront role

The evolving diagnostic landscape of bladder cancer has increasingly gravitated towards the burgeoning potential of ncRNAs as primary molecular markers (8, 14, 35). Propelled by advancements in molecular biology and oncogenomic, this shift is anchored in the substantial roles ncRNAs assume at the cellular stratum (36). Functioning beyond mere transcriptional regulators, ncRNAs have been identified to modulate diverse signaling pathways, impact gene expression, and partake in DNA repair mechanisms, highlighting their multifarious role in tumorigenesis (37–39).

Our study, through the assessment of ncRNA diagnostic accuracy, highlighted its significance in the early detection of bladder cancer, where a notable area under the curve (AUC) indicates high sensitivity and specificity. Additionally, through metrics such as positive likelihood ratio (PLR), negative likelihood ratio (NLR), and diagnostic odds ratio, we have reinforced the rigor and reliability of these findings. Compared to other diagnostic methods in bladder cancer management, ncRNA-based detection techniques demonstrate significant advantages. Traditional bladder cancer diagnostic methods, such as urine cytology and cystoscopy, despite their widespread clinical use, have certain limitations (40); urine cytology shows lower sensitivity for low-grade tumors, while cystoscopy, an invasive procedure, may cause discomfort to patients. In contrast, ncRNA-based detection methods, due to their non-invasiveness, high sensitivity, and specificity, show greater potential in early diagnosis and disease monitoring. Nonetheless, the practical application of these emerging technologies still faces challenges, including cost-effectiveness, technical standardization, and validation. As we continually unravel the intricate mechanisms via which ncRNAs function, it becomes conceivable to envisage a horizon where these entities transition from research confines to pervasive clinical applications, augmenting diagnostic precision and guiding tailored therapeutic approaches. Within the continuum of bladder cancer research, pivoting towards an understanding and leveraging of ncRNAs could well represent the next vanguard, melding molecular insights with tangible clinical implications.

### 4.3 Limitations and future directions

While this investigation offers pivotal insights, certain limitations necessitate acknowledgment. Firstly, the relatively limited number of studies included, especially those with significantly low sample sizes, could restrict the broad applicability and external validity of our conclusions. Moreover, inherent to any meta-analysis, is the latent risk of publication bias, although our evaluations suggest its minimal presence. Concerning the sources of heterogeneity, the biological heterogeneity of bladder cancer itself cannot be overlooked. Tumors in different patients may exhibit substantial differences in molecular characteristics, genetic backgrounds, and responses to treatment, which could be reflected in the variability of ncRNA expression patterns. Additionally, the differences in the sensitivity, specificity, and quantification capabilities of ncRNA detection and quantification methods might significantly contribute to the observed heterogeneity in study outcomes. Given the preliminary nature of our findings, future research should incorporate more high-quality studies to explore the role of ncRNAs in various bladder cancer subtypes, their potential value in disease prognosis, and how these molecular markers can be more effectively utilized for disease management through emerging technologies.

## 5 Conclusion

This meta-analysis accentuates the prognostic potential of exosomal ncRNAs in bladder cancer, elevated ncRNA expressions correlate with enhanced survival outcomes, advocating for their assimilation into diagnostic and therapeutic frameworks. Further research is imperative to fully capitalize on the potential of ncRNAs in the management of bladder cancer.

## Data availability statement

The original contributions presented in the study are included in the article/**Supplementary Material**. Further inquiries can be directed to the corresponding author.

## Author contributions

YC: Conceptualization, Data curation, Investigation, Methodology, Software, Writing – review & editing. KS: Data curation, Formal analysis, Methodology, Project administration, Supervision, Writing – original draft. XF: Data curation, Formal analysis, Methodology, Supervision, Writing – original draft. HG: Formal analysis, Funding acquisition, Project administration, Resources, Validation, Visualization, Writing – original draft. TG: Data curation, Formal analysis, Methodology, Software, Supervision, Writing – original draft. HY: Writing – original draft, Writing – review & editing, Conceptualization, Data curation, Formal analysis, Investigation, Methodology, Software, Supervision.
