# Supplementary material for: Diagnostic and prognostic potential of exosome non-coding RNAs in bladder cancer: a systematic review and meta-analysis
Source: Front Oncol. 2024 Mar 4;14:1336375. doi: 10.3389/fonc.2024.1336375 (PMC10944871; doi:10.3389/fonc.2024.1336375)
Supplement: Supplementary file 1 [file DataSheet_1.doc]

**Supplementary Materials**

**1 Research Strategy**

**(1) PubMed (527 items)**

#1 (((((non-coding RNA[MeSH Terms]) OR (ncRNA[MeSH Terms])) OR (long non-coding RNA[MeSH Terms])) OR (lncRNA[MeSH Terms])) OR (circRNA[MeSH Terms])) OR (miRNA[MeSH Terms])

#2: ("Bladder Cancer"[Title/Abstract] OR "Urothelial Carcinoma"[Title/Abstract])

#3: ((prognosis[MeSH Terms]) OR (outcome[MeSH Terms])) OR (survival[MeSH Terms])

#4: ((#2) AND (#3)) AND (#5)

**(2) Web of Science (1036 items)**

(TI=("Bladder Cancer" OR "Urothelial Carcinoma") AND TI=("Non-coding RNA" OR "ncRNA" OR "miRNA" OR "lncRNA") AND TI=("Prognosis" OR "Survival Rate"))

**(3) Willey Library (90 items)**

"Bladder Cancer" OR "Urothelial Carcinoma"" in Abstract and ""Non-coding RNA" OR "ncRNA" OR "miRNA" OR "lncRNA"" in Abstract and ""Prognosis" OR "Survival Rate"" anywhere

**(4) EMBASE (1159 items)**

('bladder cancer'/exp OR 'bladder cancer' OR 'urothelial carcinoma'/exp OR 'urothelial carcinoma') AND ('non-coding rna'/exp OR 'non-coding rna' OR 'ncrna'/exp OR 'ncrna' OR 'mirna'/exp OR 'mirna' OR 'lncrna'/exp OR 'lncrna') AND ('prognosis'/exp OR 'prognosis' OR 'survival rate'/exp OR 'survival rate') AND (' exosome'/exp OR ' exosome' OR ' exosomal '/exp OR ' exosomal')

**(5) Cochrane Library (9 items)**

("Bladder Cancer" OR "Urothelial Carcinoma") AND ("Non-coding RNA" OR "ncRNA" OR "miRNA" OR "lncRNA") AND ("Prognosis" OR "Survival Rate") in Title Abstract Keyword

**2 Supplemental Table**

**Table S1 Characteristics of literatures included in the meta-analysis.**

| Author | Year | Country | ncRNAs profile | Expression level | Exosomes source | Sample size  (case/control) | Exosomes Isolation and size/morphology analysis | Exosomes Positive surface makers | Exosomes Negative surface makers | ncRNAs  isolation | ncRNAs  detection assay | ncRNAs  primer sequence |
| --- | --- | --- | --- | --- | --- | --- | --- | --- | --- | --- | --- | --- |
| Abbastabar | 2020 | Iran | lncRNA ANRIL lncRNA PCAT-1 | up | Urine | 30/10 | Ultracentrifugation, TEM, NTA, 60-190nm | CD63 | Not described | Isolation kit | qRT-PCR | Yes |
| Bian | 2022 | China | lncRNA MKLN1-AS | up | Urine | 43/43 | Ultracentrifugation, TEM, NTA, 50-200nm | CD63, CD81, HSP70 | Not described | Isolation kit | qRT-PCR | Yes |
| Chen | 2020 | China | lncRNA LNMAT2 | up | Urine | 206/120 | Ultracentrifugation, TEM, NTA, 100-150nm | CD9, CD63 | Not described | TRIZOL | qRT-PCR | Yes |
| Chen | 2022 | China | lncRNA NMP-22 | up | Urine | 128/94 | Ultracentrifugation, TEM, NTA, 50-200nm | CD9, AnnexinV, TSG101, HSP70 | Not described | TRIZOL | qRT-PCR | Yes |
| Chen | 2021 | China | lncRNA ELNAT1 | up | Urine | 242/242 | Ultracentrifugation, TEM, NTA, 30-150nm | CD9, ALX, β-actin | Not described | TRIZOL | qRT-PCR | Yes |
| Chen | 2018 | China | circPRMT5 | up | Serum | 71/36 | Ultracentrifugation, TEM, NTA, Not described | CD63, TSG101, HSP70, ALX | Not described | Isolation kit | qRT-PCR | Yes |
| EL-Shal | 2022 | Egypt | miR-96-5p miR-183-5p | up | Urine | 51/28 | Isolation kit, TEM, 30-90nm | CD63, β-catenin | Not described | Isolation kit | qRT-PCR | Yes |
| Güllü Amuran | 2020 | Turkey | miR-136-3p | up | Urine | 105/34 | Ultracentrifugation, TEM, NTA, Not described | Not described | Not described | Isolation kit | qRT-PCR | Yes |
| Huang | 2021 | China | lncGAS5↓ | Down | Serum | 80/80 | Isolation kit, TEM, NTA,78.1±212.6nm | CD9, TSG101 | Not described | Isolation kit | qRT-PCR | Yes |
|  |  |  | LncMIR205HG | up | Serum | 80/80 | Isolation kit, TEM, NTA,78.1±212.6nm |  | Not described | Isolation kit | qRT-PCR | Yes |
| Lin | 2021 | China | miR-93-5p  miR-516a-5p | up | Urine | 53/51 | Ultracentrifugation, TEM, NTA, 50-200nm | CD63, TSG101 | Calnexin | TRIZOL | qRT-PCR | Yes |
| Wang | 2018 | China | LncH19 | up | Serum | 52/52 | Isolation kit, TEM, NTA, 30-150nm | TSG101, HSP70 | Not described | Isolation kit | qRT-PCR | Yes |
| Xue | 2017 | China | LncUCA1↑ | up | Serum | 30/30 | Ultracentrifugation, TEM, NTA, 50-200nm | CD63, TSG101, HSP70, HSP90 | Not described | TRIZOL | qRT-PCR | Yes |
| Yazarlou | 2018 | Iran | lncRNA UCA1-203 lncRNA UCA1-201 | up  Down | Urine | 59/49 | Isolation kit, TEM, NTA, 50-100nm | Not described | Not described | Isolation kit | qRT-PCR | Yes |
| Zheng | 2021 | China | LncBCYRN1 | up | Urine | 210/112 | Ultracentrifugation, TEM, 30-150nm | CD81, TSG101 | Not described | TRIZOL | qRT-PCR | Yes |
| Zhan | 2018 | China | lncRNA panel (MALAT1, PCAT-1, SPRY4-IT1) | up | Urine | 80/80 | Ultracentrifugation, TEM, NTA, 60-150nm | CD9, TSG101 | Not described | TRIZOL | qRT-PCR | Yes |
| Zhang | 2019 | China | lncRNA panel (PCAF1, UBC1, SNHG16) | up | Serum | 160/160 | Isolation kit, TEM, NTA, 50-100nm | CD9, TSG101 | Not described | Isolation kit | qRT-PCR | Yes |
| Zheng | 2018 | China | lncRNA PTENP1 | down | Serum | 50/60 | Isolation kit, TEM, NTA,50-120nm | CD63, TSG101 | Not described | TRIZOL | qRT-PCR | Yes |

**Table S2 The main characteristics of included literatures for prognosis analysis**

| Author | Year | Country | ncRNAs profile | TP | FP | TN | FN | Sensitivity% | Specificity% | AUC |
| --- | --- | --- | --- | --- | --- | --- | --- | --- | --- | --- |
| Abbastabar | 2020 | Iran | lncANRIL | 14 | 1 | 9 | 16 | 46.67 | 87.5 | 0.722 |
|  |  |  | lncPCAT-1 | 13 | 1 | 9 | 17 | 43.33 | 87.5 | 0.729 |
| Bian | 2022 | China | lncMKLN1-AS | 21 | 4 | 22 | 39 | 79.07 | 67.44 | 0.798 |
| Chen | 2022 | China | lncNMP-22 | 40 | 88 | 3 | 91 | 96.80 | 31.25 | 0.861 |
| El-Shal | 2021 | Egypt | miR-96-5p | 42 | 2 | 9 | 26 | 80.40 | 91.8 | 0.850 |
|  |  |  | miR-183-5p | 41 | 2 | 10 | 26 | 78.4 | 81.6 | 0.830 |
| Güllü Amuran | 2020 | Turkey | miR-136-3p | 7 | 1 | 3 | 9 | 80.0 | 88.0 | 0.899 |
| Huang | 2021 | China | LncGAS5 | 63 | 23 | 17 | 48 | 77.3 | 75.0 | 0.843 |
|  |  |  | LncMIR205HG | 62 | 14 | 18 | 66 | 78.7 | 83.1 | 0.729 |
| Lin | 2021 | China | miR-96-5p+miR-516a-5p | 42 | 3 | 11 | 48 | 85.2 | 82.4 | 0.867 |
| Wang | 2018 | China | LncH19 | 44 | 15 | 6 | 35 | 87.0 | 70.6 | 0.870 |
| Xue | 2017 | China | LncUCA1 | 24 | 5 | 6 | 25 | 80.0 | 83.3 | 0.878 |
| Yazarlou | 2018 | Iran | LncUCA1-201 | 45 | 0 | 14 | 49 | 86.0 | 55.1 | 0.730 |
|  |  |  | LncUCA1-203 | 43 | 12 | 16 | 37 | 73.5 | 73.5 | 0.660 |
|  |  |  | LncMALAT1 | 38 | 8 | 21 | 41 | 62.1 | 69.4 | 0.650 |
|  |  |  | LINC00355 | 40 | 10 | 19 | 39 | 68.0 | 79.2 | 0.750 |
| Zhan | 2018 | China | LncMALAT1 | 63 | 26 | 17 | 54 | 74.1 | 84.2 | 0.844 |
|  |  |  | LncPCAT-1 | 57 | 16 | 23 | 64 | 72.1 | 81.7 | 0.832 |
|  |  |  | LncSPRY4-IT1 | 70 | 28 | 10 | 52 | 66.3 | 76.9 | 0.760 |
| Zhang | 2019 | China | lncRNA panal | 128 | 40 | 32 | 120 | 85.7 | 78.0 | 0.878 |
| Zheng | 2018 | China | LncPTENP1 | 33 | 9 | 17 | 51 | 65.4 | 84.2 | 0.743 |

Note: （1）The table above presents the true positive (TP), false positive (FP), true negative (TN), and false negative (FN) values for ncRNA, Sensitivity (or the true positive rate) is calculated as TP / (TP + FN), and Specificity (or the true negative rate) is calculated as TN / (TN + FP). （2）The Area Under the Curve (AUC) for both models is to be determined (TBD) as more data or specialized tools are needed for its calculation.

**Table S3 Included literatures Quality evaluation (NOS Scale)**

| Author | Year | Selection | | | | Comparability  Item 5 | | Exposure | | | **Total score** | **Quality** |
| --- | --- | --- | --- | --- | --- | --- | --- | --- | --- | --- | --- | --- |
|  |  | Item 1 | Item 5 | Item 3 | Item 4 |  |  | Item 6 | Item 7 | Item 8 |  |  |
| Abbastabar | 2020 | 1 | 1 | 1 | 1 | 1 | 1 | | 0 | 1 | 7 | Good |
| Bian | 2022 | 0 | 1 | 1 | 0 | 1 | 1 | | 1 | 0 | 6 | Good |
| Chen | 2020 | 1 | 1 | 1 | 0 | 1 | 1 | | 1 | 1 | 6 | Good |
| Chen | 2022 | 0 | 1 | 1 | 0 | 1 | 0 | | 1 | 1 | 5 | Moderate |
| Chen | 2021 | 1 | 1 | 1 | 0 | 1 | 0 | | 1 | 1 | 6 | Good |
| Chen | 2018 | 1 | 1 | 1 | 0 | 1 | 0 | | 1 | 1 | 6 | Good |
| El-Shal | 2021 | 1 | 1 | 1 | 0 | 0 | 1 | | 1 | 1 | 6 | Good |
| Güllü Amuran | 2020 | 1 | 1 | 1 | 0 | 1 | 1 | | 1 | 1 | 7 | Good |
| Huang | 2021 | 0 | 1 | 1 | 0 | 1 | 0 | | 1 | 1 | 5 | Moderate |
| Lin | 2021 | 1 | 1 | 1 | 0 | 1 | 1 | | 0 | 1 | 6 | Good |
| Wang | 2018 | 0 | 0 | 1 | 0 | 1 | 1 | | 1 | 1 | 5 | Moderate |
| Xue | 2017 | 1 | 0 | 1 | 0 | 1 | 1 | | 1 | 1 | 6 | Good |
| Yazarlou | 2018 | 1 | 1 | 1 | 0 | 1 | 0 | | 1 | 1 | 6 | Good |
| Zhan | 2018 | 1 | 1 | 1 | 0 | 1 | 0 | | 1 | 1 | 6 | Good |
| Zhang | 2019 | 1 | 1 | 1 | 0 | 0 | 1 | | 1 | 1 | 6 | Good |
| Zheng | 2021 | 1 | 1 | 1 | 0 | 1 | 1 | | 1 | 1 | 8 | Good |
| Zheng | 2018 | 1 | 1 | 1 | 0 | 0 | 1 | | 1 | 1 | 6 | Good |

Note: Item 1: Representativeness of the exposed cohort; item 2: Selection of the non-exposed cohort; item 3: Ascertainment of exposure; item 4: Demonstration that outcome of interest was not present at start of study; item 5: Comparability of cohorts on basis of the design or analysis; item 6: Assessment of outcome; item 7: Was follow up long enough for outcomes to occur; item 8: Adequacy of follow up of cohorts.

**Table S4 Sensitivity analysis of OS**

| study | 95%-CI | p-value | tau^2 | tau | I^2 |
| --- | --- | --- | --- | --- | --- |
| Omitting Chen 2022 | 1.0813 [0.9203; 1.2705] | 0.3417 | 0.2537 | 0.5037 | 88.50% |
| Omitting Zheng 2021 | 1.0540 [0.8934; 1.2436] | 0.5329 | 0.2475 | 0.4975 | 87.90% |
| Omitting Chen 2020 | 1.2501 [1.0594; 1.4752] | 0.0082 | 0.1577 | 0.3971 | 84.50% |
| Omitting Chen 2021 | 1.0321 [0.8738; 1.2190] | 0.7103 | 0.2317 | 0.4814 | 87.00% |
| Omitting Chen 2021 | 1.0247 [0.8673; 1.2105] | 0.7746 | 0.2249 | 0.4743 | 86.60% |
| Omitting Chen 2018 | 1.3477 [1.1302; 1.6070] | 0.0009 | 0.1559 | 0.3948 | 79.90% |
| Pooled estimate | 1.1214 [0.9631; 1.3057] | 0.1402 | 0.212 | 0.4605 | 86.30% |

**Table S4 Sensitivity analysis of DFS**

| study | 95%-CI | p-value | tau^2 | tau | I^2 |
| --- | --- | --- | --- | --- | --- |
| Omitting Chen 2022 | 1.1328 [0.9892; 1.2973] | 0.0715 | 0.1531 | 0.3913 | 76.50% |
| Omitting Zheng 2021 | 1.1282 [0.9810; 1.2974] | 0.0909 | 0.1639 | 0.4048 | 77.00% |
| Omitting Chen 2020 | 1.3002 [1.1283; 1.4984] | 0.0003 | 0.107 | 0.3272 | 69.50% |
| Omitting Chen 2021 | 1.1295 [0.9811; 1.3003] | 0.0902 | 0.1656 | 0.4069 | 77.20% |
| Omitting Chen 2021 | 1.0993 [0.9548; 1.2657] | 0.1878 | 0.1556 | 0.3944 | 75.10% |
| Omitting Chen 2018 | 1.3646 [1.1796; 1.5786] | < 0.0001 | 0.0889 | 0.2981 | 60.10% |
| Omitting Zhan 2018 | 1.1755 [1.0309; 1.3405] | 0.0158 | 0.1545 | 0.3931 | 78.10% |
| Omitting Zhan 2018 | 1.1650 [1.0217; 1.3284] | 0.0225 | 0.1506 | 0.388 | 77.80% |
| Omitting Zhan 2018 | 1.1685 [1.0248; 1.3324] | 0.0201 | 0.1539 | 0.3923 | 78.10% |
| Omitting Zhang 2019 | 1.1642 [1.0193; 1.3296] | 0.0249 | 0.1606 | 0.4007 | 78.00% |
| Omitting Zhang 2019 | 1.1436 [1.0017; 1.3057] | 0.0472 | 0.1366 | 0.3696 | 75.90% |
| Omitting Zhang 2019 | 1.1560 [1.0122; 1.3202] | 0.0324 | 0.1553 | 0.394 | 77.60% |
| Pooled estimate | 1.1730 [1.0296; 1.3363] | 0.0164 | 0.146 | 0.3821 | 76.00% |

**3 Supplemental Figure**

**
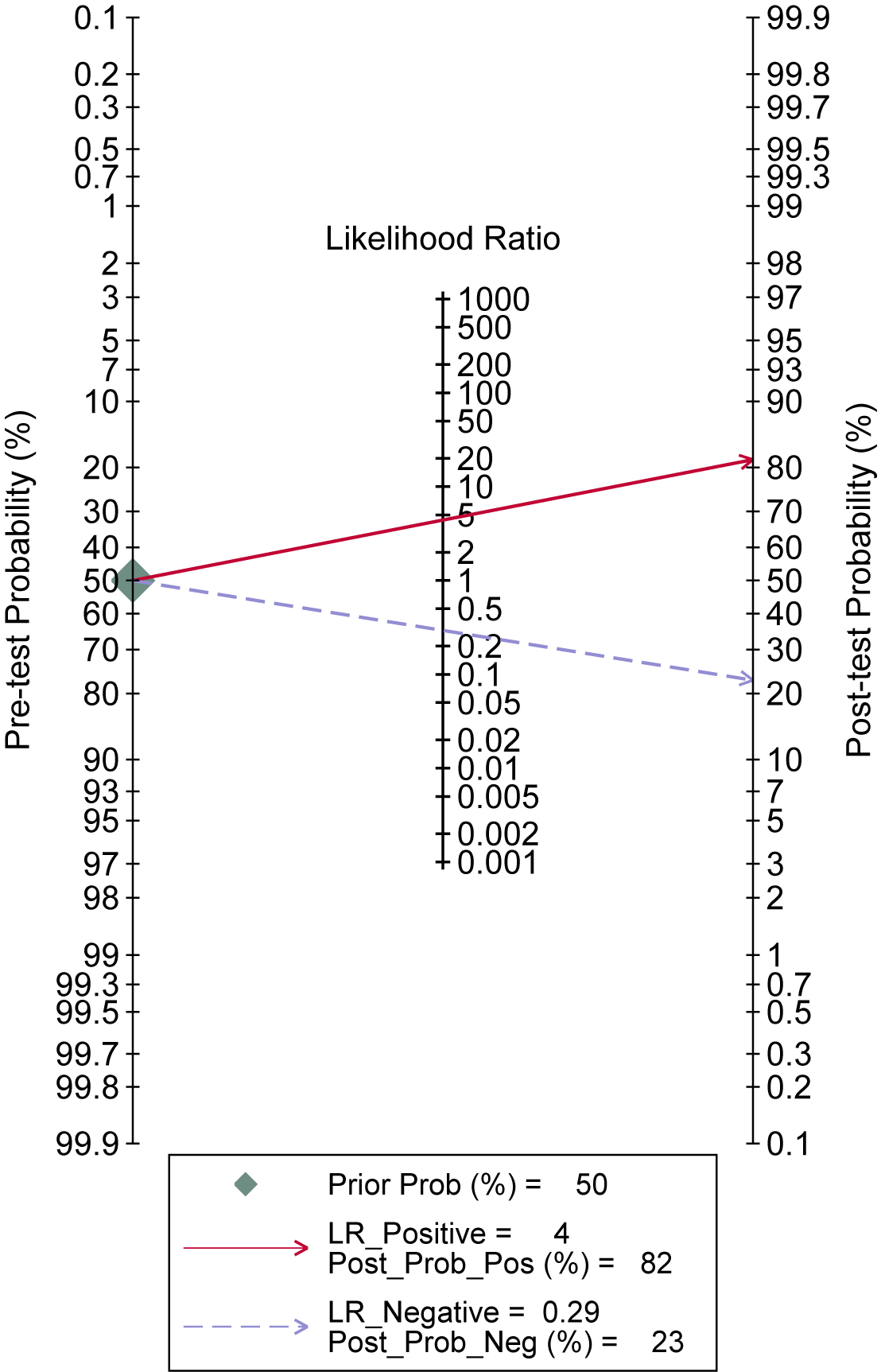
**

**Figure S1 Faggn for Figure prior probability and posterior probability**

**
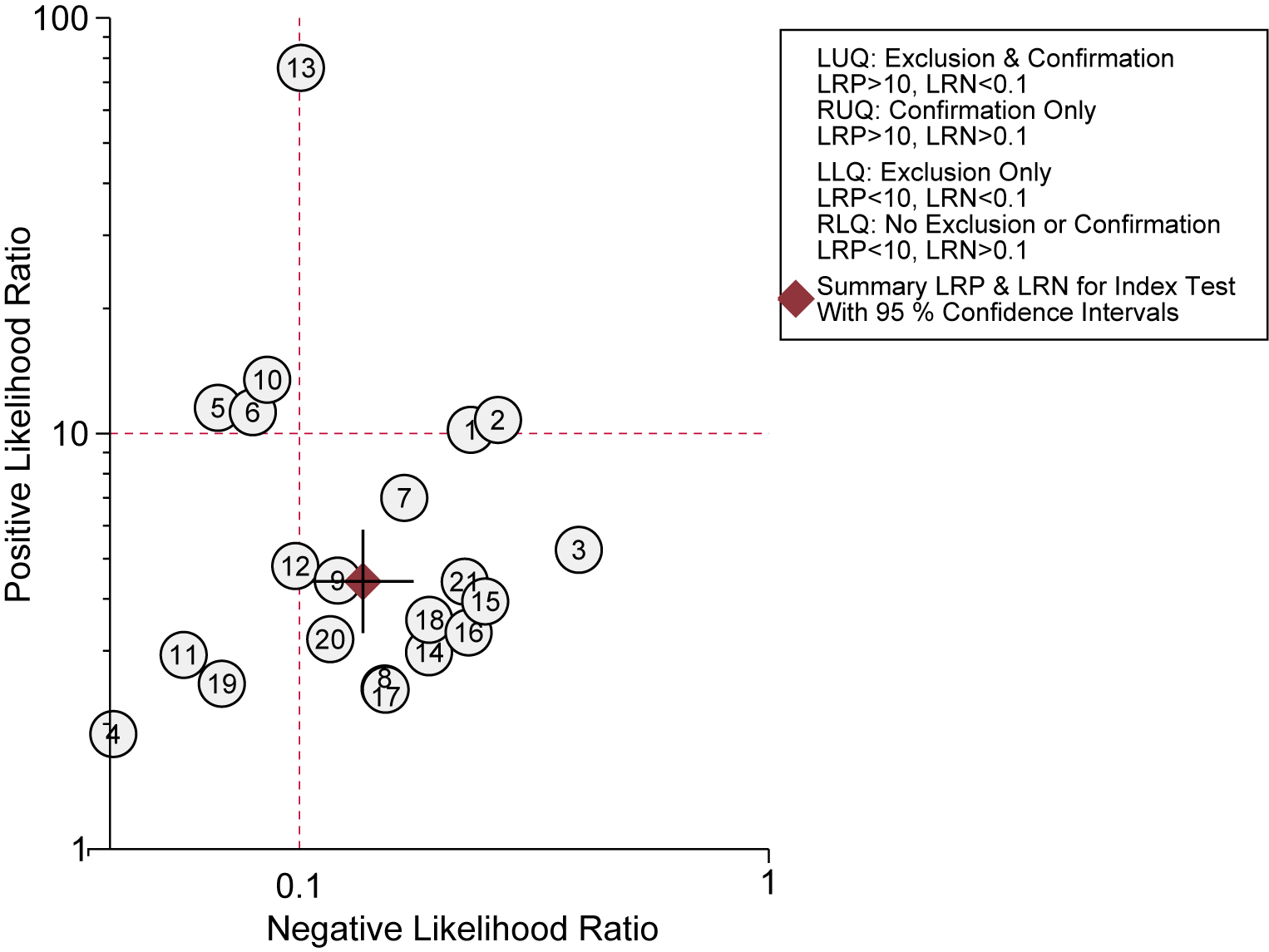
**

**Figure S2. Distribution scatter diagram**
